# Characteristics of Micro-Seismic Events Induced by Ground Collapse—A Case Study in the Rongxing Gypsum Mine in Hubei Province, China

**DOI:** 10.3390/s24041309

**Published:** 2024-02-18

**Authors:** Hongzhu Wang, Taiyi Chen, Guangli Xu

**Affiliations:** Faculty of Engineering, China University of Geosciences, Wuhan 430074, China; whz@cug.edu.cn (H.W.); chentaiyi1218@cug.edu.cn (T.C.)

**Keywords:** micro-seismic event, ground collapse, waveform characteristic, F-T-A model, gypsum mine

## Abstract

Mining activities can damage rock masses and easily induce ground collapse, which seriously threatens safe production in mining areas. Micro-seismic systems can monitor rock mass deformation signals in real time and provide more accurate data for rock mass deformation analysis. Therefore, in this study, the waveform characteristics of micro-seismic events induced by ground collapse in the Rongxing gypsum mine were analyzed; the occurrence of these events was introduced on the basis of Fast Fourier Transform, an established Frequency–Time–Amplitude model, in order to put forward the index of energy proportion of the main band. The results showed the following. (1) The seismic sequence type of ground collapse was foreshock–mainshock–aftershocks. The interval between the foreshock and mainshock was longer than that between the mainshock and aftershocks. (2) The deformation corresponding to the foreshock micro-seismic events was mainly that of a small-scale crack. The deformation corresponding to the micro-seismic events during the mainshock was characterized by the gradual development of small-scale cracks, and the development of large-scale cracks accelerated, accompanied by slight rock collapse. The deformation corresponding to the micro-seismic events during the aftershocks showed that almost no small-scale cracks developed, and the large-scale crack development was intense, and accompanied by numerous rock and soil mass collapses. (3) The observed decreasing frequency distribution and energy dispersion can be used as possible precursors of ground collapse.

## 1. Introduction

The gypsum mineral resources are very rich in China, and most of them are present in sedimentary deposits. The roofs of gypsum mines generally consist of argillaceous sandstone or mudstone with low strength [1,2]. In addition, most gypsum mines have been excavated using the room-and-pillar method. Insufficient early management and indiscriminate mining have resulted in large and irregular gypsum mine goafs, and in turn have caused large-scale ground collapse [3,4,5]. For example, in November 2005, the Xingtai gypsum mine in China suffered a massive crown collapse, resulting in many casualties, 37 deaths and 38 injuries [6].

Due to the complex geological conditions and irregular characteristics of mining areas [7,8,9], research on ground collapse has been limited. The accuracy and timeliness of mature monitoring equipment, such as interferometric synthetic aperture radar (InSAR) and global navigation satellite system (GNSS), are also insufficient. The process of ground collapse deformation is mostly analyzed by surface deformation, while underground rock deformation is poorly understood [10,11,12]. Therefore, the development of stable and time-efficient monitoring technology for mining ground collapse has always been a research hotspot.

Under the action of geo-stress, the rocks in a goaf undergo deformation, fracturing, and potential collapse. During this process, the rock mass releases strain energy to the external environment in the form of elastic waves, which is called micro-seismic (MS) activity [13,14]. A highly sensitive detector can automatically collect MS information about the rock cracking process. The information can be recorded, processed, and analyzed through software, allowing for the inference and analysis of various seismic characteristics, including the time, location, energy, and other characteristics of the MS events [15,16]. This technology is called MS monitoring technology.

Considering the advantages of high sensitivity, real-time monitoring, and dynamic data, MS monitoring technology has been utilized in mining area monitoring by many scholars, and noteworthy research outcomes have been achieved. Li et al. [17] conducted an in-depth investigation into the characteristics of MS waves and found that the characteristics of impact MS waves could be used to effectively identify and extract valuable precursor information for rock dynamic disaster prediction. This research provides a method for predicting rock dynamic disasters by analyzing the characteristics of MS waves. Liu et al. [18] developed an MS event detection method based on a convolutional neural network (CNN) that could quickly and effectively classify monitoring data and greatly improve the efficiency of MS event identification. Leng et al. [19] proposed a global optimization algorithm based on waveform similarity coefficients and iterative cross-correlation to refine the arrival time of MS waves and improve the longitude of the source location. Li et al. [20] monitored and analyzed a high-intensity mining coal mine, and the results showed that the parameter characteristics of MS events were obviously abnormal before and after rock destruction, and that the energy release of MS events depended on the pressure. Li et al. [21] investigated the dynamic response characteristics of MS events such as blasting mining, coal outflow, and coal wall collapse. The results showed that the low-frequency part of the MS signals was mainly caused by rock damage, and the high-frequency part was caused by rock collapse.

Time–frequency analysis is a mathematical analysis method that can convert micro-seismic waves from the time domain to the frequency domain [22]. By analyzing the frequency domain, the frequency domain characteristics of micro-seismic events can be obtained. As the most traditional time–frequency analysis method, the Fast Fourier Transform is widely used in signal analysis.

Benson et al. [23] used the FFT to process the waveforms of basalt deformation and fracture under 40 MPa of confining pressure, and the results showed that the proportion of volcanic–tectonic seismic or low-frequency events in mixed events depends on the pore fluid present in the rock type and the degree of rock failure. Chernov et al. [24] proposed a method for estimating the seismic intensity on the MMI or MSK scale based on the Fourier amplitude spectrum of ground acceleration, estimated the probable field intensity from seismic records in several seismic regions, and predicted the intensity distribution patterns of certain earthquakes.

Before a rock burst occurred, the frequency of the MS signal was low, and the frequency tended to increase to a low frequency. When a rock burst occurred, the MS signal exhibited low-frequency characteristics, and the amplitude increased to the maximum value [25]. Most of the previous studies focused on the characteristic analysis of MS events induced by rock bursts in coal mines. The analysis of MS events induced by ground collapse in gypsum mines could only serve as a reference for identifying MS events. Research on MS events induced by ground collapse is lacking, and almost no research has been conducted on MS events during different periods of ground collapse. The characteristics of rock burst MS events and ground collapse MS events are essentially the characteristics of elastic waves released when a rock mass is broken. Therefore, it is feasible to use the characteristics of rock burst MS events as a reference.

In this study, we analyzed the waveform characteristics of MS events induced by ground collapse in the Rongxing gypsum mine, introduced time on the basis of the Fast Fourier Transform (FFT), and established the Frequency–Time–Amplitude (F-T-A) model. Then the changes in the frequency distribution and energy released were analyzed.

## 2. Materials and Methods

**Figure 1:**
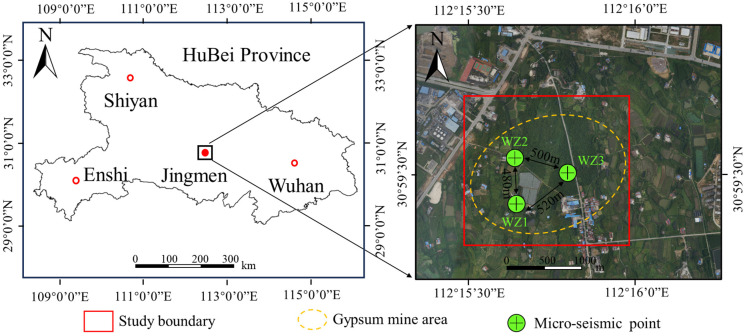
Geographical location and micro-seismic point deployment of the Rongxing gypsum mine (the yellow gypsum mine area is a reference area).

The Rongxing gypsum mine, located in Jingmen, Hubei Province (E112°15′35.70″, N30°59′33.15″), represents a typical case of ground collapse, and its geographical location is shown in Figure 1. The study area is a low-grade hilly landform, with a maximum elevation of +104.5 m, the lowest elevation of +60.3 m, a relative elevation difference of 44.2 m, and a mountain slope ranging from 5 to 10°. The ground is mainly forested, farmland, roads, residential houses, and small weirs. Three MS sensors were set in the goaf and the distance was measured.

**Figure 2:**
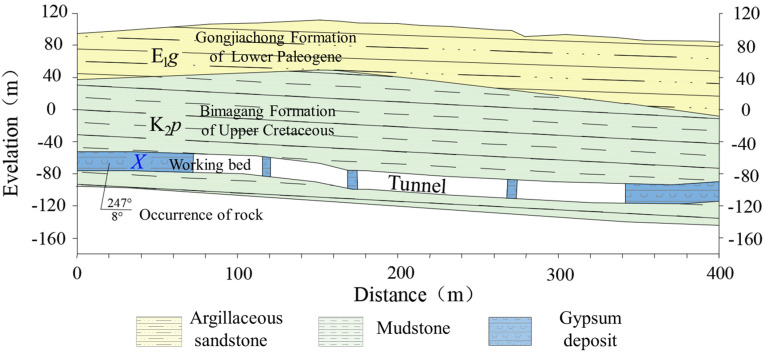
Geographical location of the Rongxing gypsum mine (exaggeration of mined ore layers to highlight gypsum deposits).

The thickness of the overburden layer in the upper part of the mining area is 35.8–238.5 m. This layer is generally thin in the northeast, thick in the southwest, locally thick in the middle, and thin in the north and south in the central part of the area, as showed in Figure 2. The thickness of the overburden layer is 95–155 m. At present, the Rongxing gypsum mine is in a state of slow subsidence after collapse. The mining layer in the study area is the Ⅹ paste layer, which is mined by the room-and-pillar method. The thickness of the layer is 1.91–12.98 m, and the elevation is characterized by high northeast and low southwest, which causes the distribution of the mining area to be characterized by high northeast and low southwest.

**Figure 3:**
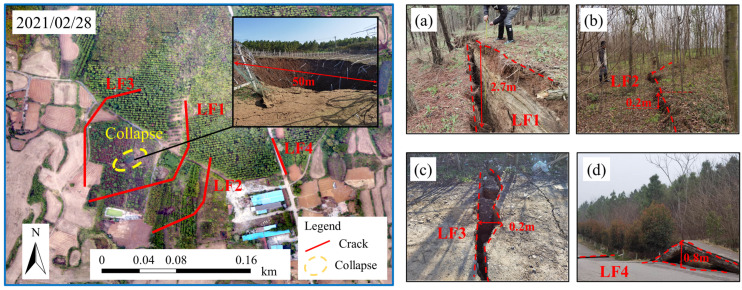
The characteristics of ground collapse. (**a**) Deformation photo of LF 1; (**b**) deformation photo of LF 2; (**c**) deformation photo of LF 3; (**d**) deformation photo of LF 4.

At approximately 10:00 a.m. on 17 February 2021, a ground collapse occurred in the Rongxing gypsum mine. The geographic coordinates of the center of the collapse area were 112°15′35.70″ E, 30°59′33.15″ N. Figure 3 shows an elliptical collapse pit in the main deformation area of the RX gypsum mine, with an area of 595.4 m^2^, a long axis of approximately 50 m, a strike direction of 40°, a short axis of approximately 30 m, and the deepest collapse of 8~10 m. The cracks developed in the periphery of the main deformation area, and the main cracks were circularly connected. The cracks on the southeast side of the collapse pit had the largest scale and were the most obvious faults (LF1 and LF2). The overall width of the cracks was 0.2–0.3 m, and the faults were 0.5–1.0 m long to form a steep slope, which was high in the east and low in the west, with a visible depth of 1.5 m. The cracks (LF3) on the north and west sides of the collapse pit were 0.1–0.2 m in width, 0.1–0.2 m in descent, high in the south, and low in the north, with a visible depth of approximately 0.5 m. The crack (LF4) on the east side of the collapse pit was approximately 3 cm wide and was mainly manifested as pavement uplift and tensile cracks.

### 2.1. The Institute of Mine Seismology Monitoring System

**Figure 4:**
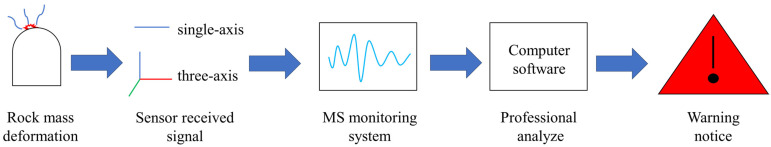
Schematic diagram of the micro-seismic monitoring system.

The development process of the gypsum mine collapse MS monitoring system is schematically shown in Figure 4. The nature of a goaf collapse is a series of rock mass rupture events that develop from the collapse source. After a rock crack occurs, the generated vibration waves spread outward through the surrounding rock. The single-axis or three-axis sensors close to the rock wall convert the original MS signals received into electrical signals, which are then transmitted to the underground data exchange center in real time, and the data signals will be sent to the central server of the MS station through the communication system. Professionals operate the analysis and processing software to extract and analyze MS data in many aspects and levels, infer the occurrence time, location, and trend of MS events and determine the collapse process of gypsum mines. Through real-time analysis of the number of MS events in gypsum mines and the spatiotemporal evolution of the corresponding focal parameters (such as energy and apparent volume), it is possible to analyze the development process of the collapse.

The Institute of Mine Seismology’s monitoring system demonstrates high positioning accuracy and is suitable for application in hard rock, medium-hard rock, and high-stress mining environments. Its primary usage extends to non-coal mines and open-pit slope monitoring. The sensors can receive MS waves with an acceleration greater than 10^−6^ m/s^−2^, and a sampling rate of 192,000 SPS. The proximity of the MS sensors to the goaf enhances signal reception effectiveness and precision. However, positioning the sensors too close to the goaf carries the risk of equipment damage due to the goaf collapse. Consequently, it is recommended to position the sensors above the goaf caving zone and fracture zone. Simultaneously, maintaining a vertical separation of 10–30 m among sensors enables monitoring across multiple strata, maximizing the effectiveness of the monitoring process. The sensor burial depths are detailed in Table 1.

### 2.2. F-T-A Model

The Fast Fourier Transform, as one of the most classical and widely used frequency analysis methods, is suitable for studying the overall distribution of waveform frequencies. The transformation formula is as follows [26,27,28].

(1) $$F(f)=\int_{-\infty }^{+\infty }x(t)e^{-j2\pi ft}dt$$

where *f*, *t*, and *j* stand for the frequency, time, and imaginary unit, respectively; *e* means the base of natural logarithms.

**Figure 5:**
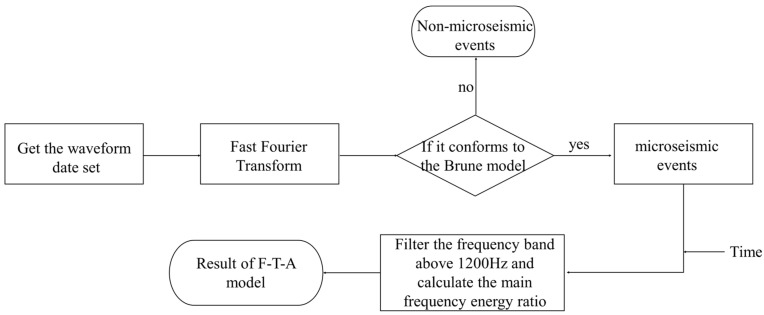
Flowchart of Frequency–Time–Amplitude model construction.

The frequency spectrum of waveforms obtained through an FFT can reflect the overall distribution of the frequency spectrum of waveforms but cannot reveal temporal information. Therefore, we introduced time on the basis of the FFT and established the F-T-A model. Figure 5 illustrates the process of the F-T-A model.

### 2.3. Ground Collapse Model

**Figure 6:**
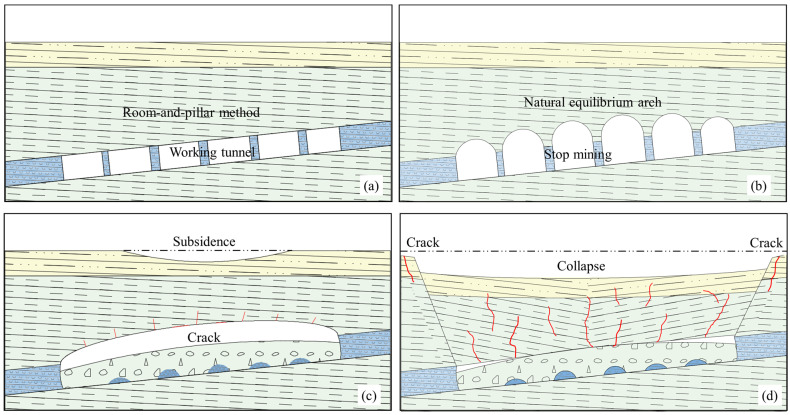
Schematic diagram of the ground collapse deformation process. (**a**) Room-and-pillar mining (man-made); (**b**) natural stress arch formation (natural); (**c**) rock beam deformation (natural); (**d**) ground collapse (natural).

According to the existing research on the mechanism of ground collapse [28,29,30,31], ground collapse in the Rongxing gypsum mine can be roughly categorized into four processes. Figure 6 illustrates the four processes. (a) Gypsum mining via the room-and-pillar method creates a mining void area that disrupts the equilibrium of the rock in its natural state. (b) When the thickness of the overlying strata in the goaf is sufficient to provide space for the expansion of the stress arch, the span of the stress arch is greatly increased, forming a support structure dominated by rock beams and maintaining a certain balance. (c) When the rock beam is bent to a certain extent, microcracks first appear in the center and corner of the roof. (d) As the overlying rock pressure continues to increase, cracks penetrate the surface, and the pillar compresses violently and begins to collapse. When the pillar is not strong enough to support the overlying rock, the roof collapses as a whole. Since the rock beam has a certain thickness, a gentle slope appears in the pit when the deformation reaches the surface. Due to the compression of the upper part of the rock beam, the local surface of this kind of collapse often experiences extrusion deformation, small compressive shear cracks are also common in the settlement area, and the outer part of the settlement area is dominated by tension cracks.

## 3. MS Monitoring Data Analysis

**Figure 7:**
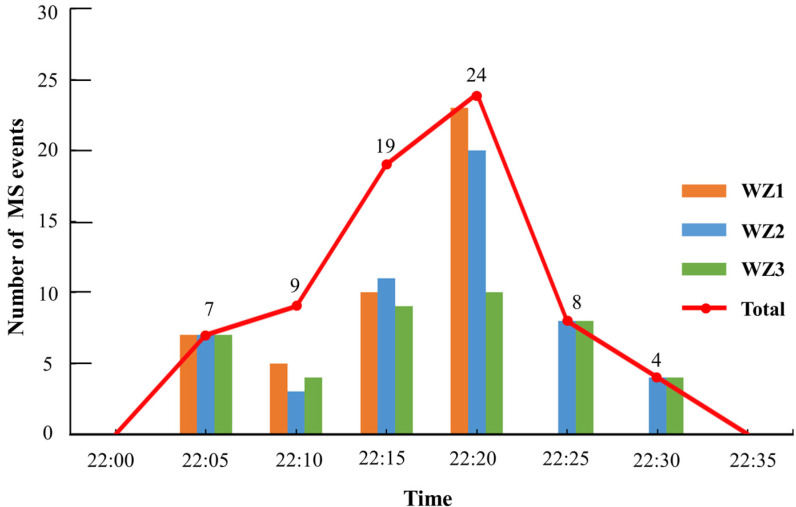
Line graph of micro-seismic events versus time.

The IMS monitoring data showed that the system monitored the first MS event at 22:01:36 on 16 February 2021, followed by a gradual increase in the number of MS events. Figure 7 and Table 2 illustrate the relationships between the number of MS events and time. The MS event manifested as a rockfall waveform fragment at 22:05:24, and the MS event decreased abruptly after 22:20:31, until it ceased at 22:26:24. 

The maximum amplitude of MS events is a crucial indicator of MS events and is often used for their classification. Since the energy is proportional to the amplitude squared, the larger the maximum amplitude received by the sensors, the greater the energy carried by the MS events. Thus, the correlation between the energy released from the mine collapse area and the process of mine collapse is reflected from the level of MS amplitude. Since the identification of S waves was complicated and the arrival time was difficult to determine precisely, P wave data were chosen for analysis.

**Figure 8:**
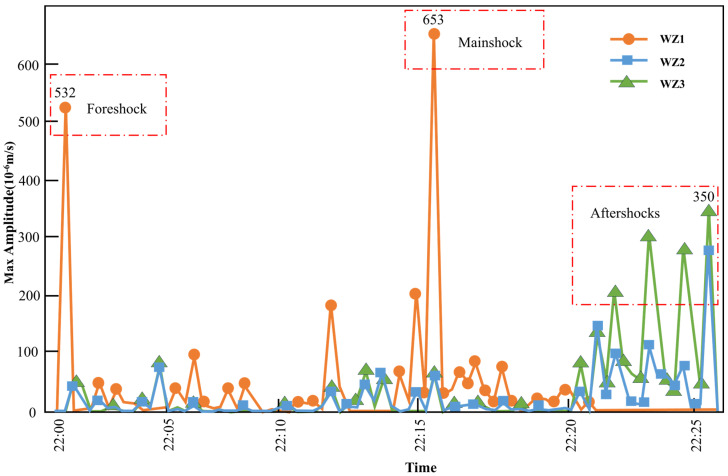
Line graph of the amplitude versus time.

The maximum amplitude of MS events received by the MS system is mostly concentrated at 10^−6^ m/s. Figure 8 illustrates the relationship between the maximum amplitude of MS events and time. At 22:00–22:10, WZ1 received MS events with the largest maximum amplitude at 22:01:36, followed by MS events with maximum amplitudes less than 5 × 10^−6^ m/s, indicating that the collapse source was closest to WZ1, and that the crack subsequently developed away from WZ1. The max amplitudes of the first MS event received by WZ2 and WZ3 were similar, indicating that there was not a large difference in the distance between WZ2 and WZ3 and the collapse source. However, WZ2 was slightly closer.

The maximum amplitude of the MS event increased at 22:10–22:20, which indicated that the deformation of the rock was more intense and that the cracks developed continuously. At 22:15:13, WZ1 received an MS event with a max amplitude of 653 × 10^−6^ m/s, indicating that severe deformation of the rock occurred at this time.

At 22:20–22:30, WZ1 did not receive MS events, and the maximum amplitudes of MS events received by WZ2 and WZ3 were concentrated at 100–300 × 10^−6^ m/s, indicating that the rock deformation was mainly near WZ2 and WZ3 at this time, and the rock mass deformation at the collapse source (near WZ1) tended to stop.

There were two higher peaks at 22:01:36 and 22:15:13, and several lower peaks between 22:20:02 and 22:26:24. Therefore, the seismic sequence of ground collapse can be defined as foreshock–mainshock–aftershocks. The maximum amplitude of the foreshock was relatively low, there were relatively few MS events, the maximum amplitude of the mainshock was large, there were relatively more MS events, the maximum amplitude of the aftershock was small, and there were also less MS events. Therefore, the precursor of ground collapse was an MS event with a large maximum amplitude.

As the monitoring data from the surface GNSS equipment showed, ground subsidence started at 23:10 on February 16, approximately 40 min after the aftershocks. The ground collapse lasted for a total of 17 h, with a slower rate of surface settlement during the first 11 h and an accelerated rate during the second 6 h.

## 4. Energy Distributions of MS Events

**Figure 9:**
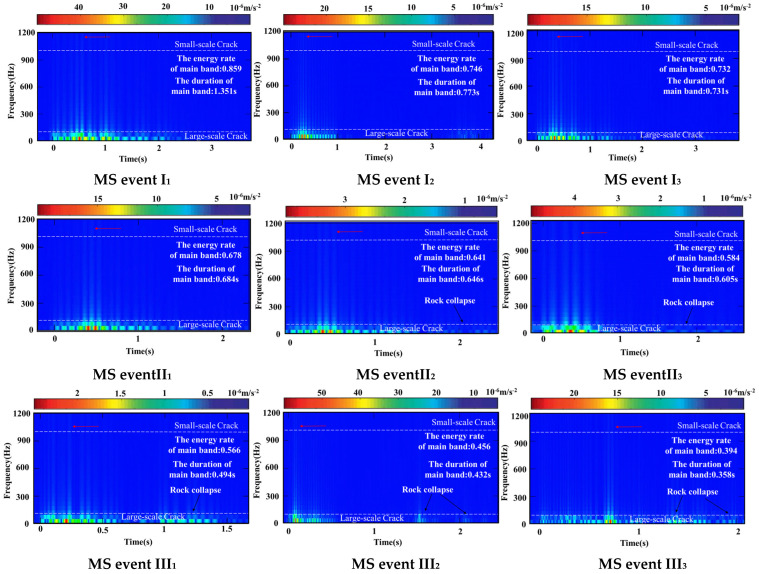
Results of the Frequency–Time–Amplitude model.

A total of nine MS events were uniformly selected near I—the foreshock, II—the mainshock, and III—the aftershock, and the changes in the deformation energy released from the rock masses of the MS events were investigated by the F-T-A model, as shown in Figure 9. A new evaluation index is proposed here—the energy ratio of the main band, that is, the ratio of the energy released by the main band in the whole MS event. The larger the proportion of the main band energy, the more concentrated the energy release, the more dispersed, and vice versa.

In I—the foreshock, the duration of the MS signals was mostly 1 s and the energy released was concentrated. Energy was released at 1000–1200 Hz, but the energy was low, indicating that the deformation corresponding to this frequency band was a small-scale crack. Energy was released only in the main frequency segment, the maximum energy was concentrated at 0–100 Hz, and the corresponding deformation was a large-scale crack. That is, in I, the deformation of the rock mass involved the development of cracks of various scales, and no rock collapse occurred. 

In II—the mainshock, the duration of the MS signals was mostly 2 s and the energy released was more dispersed. Among them, the energy decreased at 1000–1200 Hz, two maximum energy points appeared at 0–100 Hz, and the energy began to disperse. In other words, in II, the deformation of the rock and soil mass occurred because the development of small-scale cracks slowed, and the development of large-scale cracks accelerated, accompanied by the collapse of a small number of rock and soil masses. 

In III—the aftershocks, the duration of the MS signals was mostly 2 s and the energy released was dispersed. There was almost no energy released at 1000–1200 Hz, and the energy was mainly concentrated at 0–100 Hz. In III, there was almost no small-scale crack development, and large-scale crack development was intense, accompanied by numerous rock and soil mass collapses.

According to the F-T-A results, the duration of the main band continued to decrease, the frequency also continued to decrease in I-III, and the energy released was low in I-II and high in III.

## 5. Discussion

The mechanism of ground collapse in mining areas is highly complex, and the combination of multiple exploration methods, experimental data, monitoring means, and new theories from other disciplines is usually needed to elucidate the ground subsidence process [32,33,34,35].

MS signals contain a large amount of information about the ground subsidence process, with different damage modes manifesting in distinct characteristics of MS events [36,37]. As a precursor event of ground subsidence, MS events must be accurately analyzed.

According to the results, MS events exhibited better continuity across the collapse, zone, as indicated by the greater number of MS events, the sudden increase in the maximum amplitude, the lack of concentrated energy release, and the increase in frequency fluctuations. This observation provides information on the deformation process during ground collapse.

The results of the FTA model showed that the deformation corresponding to MS events was crack expansion and slight rock and soil mass collapse. Combined with the process of ground collapse, this process occurred during the (b) natural stress arch formation and (c) rock beam deformation stage. In this period, the seismic sequence type corresponding to ground collapse was foreshock–mainshock–aftershock, the time interval between the foreshock and mainshock was longer, and the time interval between the mainshock and aftershocks was shorter. In the foreshock, the cracks developed stably, and in the mainshock–aftershock phase, the fractures developed stably until the rock and soil masses were completely destroyed. In the stage from stable fracture development to unstable fracture development, with increasing rock and soil mass cracking, the frequency distribution of MS events exhibited a downward trend, and the release of energy exhibited a decentralized trend, indicating that the possibility of ground collapse increased. Therefore, the decreasing frequency distribution and energy dispersion of MS events can be used as precursors of ground collapse. If timely measures can be taken in the mining area when a precursor is detected, this approach has positive significance for ensuring the safety and progress of site construction.

Many scholars who study the time–frequency characteristics of coal mine rock burst MS events had adopted the wavelet packet transform to process the MS events [38]. This method can filter the interference of mining activities on the results to a certain extent, while retaining the precision of the main frequency segment of MS events. For gypsum mines, due to the limited gypsum resources, the mining tunnel is shallow and the ground pressure is low, so the possibility of rock burst is little. However, due to the unknown location and depth of the tunnel excavated in history, once a gypsum mine collapses, other surrounding gypsum mines will also be closed. Therefore, it is not necessary to consider the interference of mining activities when analyzing the existing ground collapse MS events. Using FFT to process ground collapse MS events, the accuracy of the frequency domain is retained to a great extent. In the time–frequency analysis, the possibility of ground collapse can be judged by the proportion of the main band energy in the FTA model results. Of course, this method is currently only applicable to gypsum mines without interference from mining activities. At present, we are exploring a method that can filter the interference of mining activities and ensure the precision of MS events, with the aim of real-time early warning of ground collapse.

## 6. Conclusions

Elastic waves were generated when the underground rock mass was deformed, and the elastic waves were recorded by the IMS monitoring system. Firstly, the relationships between the number, frequency, and maximum amplitude of MS events were analyzed. Secondly, an FFT was used to process MS signals, the time was introduced, and an FTA model was established. By analyzing the changes in the MS event frequency and energy distribution with respect to the damage degree of rock and soil mass, the following conclusions can be drawn:

(1) According to the frequency and amplitude curves of the MS events, the seismic sequence type corresponding to ground collapse was foreshock–mainshock–aftershocks, the energy released by the foreshock was high, and the number of MS events was low. The energy released by the mainshock was high, and the number of MS events was low. Aftershocks released less energy, and the number of MS events was small. The interval between the foreshock and mainshock was longer than that between the mainshock and aftershocks.
(2) The deformation corresponding to MS events in the foreshock was mainly small-scale cracks, the deformation corresponding to MS events in the mainshock mainly due to the development of small-scale cracks slowed, and the development of large-scale cracks accelerated, accompanied by slight rock collapse. The deformation corresponding to MS events during the aftershocks showed that almost no small-scale cracks developed, and the large-scale crack development was intense, accompanied by numerous rock and soil mass collapses.
(3) A decreasing frequency distribution and energy dispersion can be used as precursors of ground collapse.

Based on the FFT, time was introduced to establish an FTA model for rock mass deformation analysis, which provides a reference for ground collapse warnings.

## Figures and Tables

**Table 1:** The depth of micro-seismic sensors.

Number	Depth/m
WZ1	70
WZ2	90
WZ3	80

**Table 2:** The number of MS events.

Time	The Number of MS Events			
	WZ1	WZ2	WZ3	Total
22:00–22:05	7	7	7	7
22:05–22:10	5	3	4	9
22:10–22:15	10	11	9	19
22:15–22:20	23	20	10	24
22:20–22:25	0	8	8	8
22:25–22:30	0	4	4	4

## Data Availability

The data presented in this study are available upon request from the corresponding author.
